# Psychobiological Changes during National Futsal Team Training Camps and Their Relationship with Training Load

**DOI:** 10.3390/ijerph17061843

**Published:** 2020-03-12

**Authors:** Filipe Manuel Clemente, Ana Filipa Silva, Hugo Sarmento, Rodrigo Ramirez-Campillo, Yi-Wen Chiu, Yu-Xian Lu, Pedro Bezerra, Yung-Sheng Chen

**Affiliations:** 1 Escola Superior Desporto e Lazer, Instituto Politécnico de Viana do Castelo, Rua Escola Industrial e Comercial de Nun’Álvares, 4900-347 Viana do Castelo, Portugal; anafilsilva@gmail.com (A.F.S.); pbezerra@esdl.ipvc.pt (P.B.); 2 Instituto de Telecomunicações, Delegação da Covilhã, 1049-001 Lisboa, Portugal; 3 N2i, Polytechnic Institute of Maia, 4475-690 Maia, Portugal; 4 The Research Centre in Sports Sciences, Health Sciences and Human Development (CIDESD), 5001-801 Vila Real, Portugal; 5 Research Unit for Sport and Physical Activity (CIDAF), Faculty of Sport Sciences and Physical Education, University of Coimbra, Coimbra, 3040-156 Coimbra, Portugal; hg.sarmento@gmail.com; 6 Human Performance Laboratory. Quality of Life and Wellness Research Group. Department of Physical Activity Sciences, Universidad de Los Lagos. Lord Cochrane 1046, Osorno, Chile; r.ramirez@ulagos.cl; 7 Centro de Investigación en Fisiología del Ejercicio. Facultad de Ciencias. Universidad Mayor. Santiago, Av Libertador Bernardo O’Higgins 2027, Chile; 8 Department of Physical Education, Fu Jen Catholic University, New Taipei 24205, Taiwan; 055332@mail.fju.edu.tw; 9 Department of Exercise and Health Sciences, University of Taipei, Taipei 11153, Taiwan; whitelu0422@gmail.com (Y.-X.L.); yschen@utaipei.edu.tw (Y.-S.C.); 10 Graduate Institute of Athletes and Coaching Science, National Taiwan Sport University, Taoyuan 33301, Taiwan

**Keywords:** youth, performance, workload, physiological phenomena, medical psychology, heart rate

## Abstract

The aim of this study was two-fold: (1) to analyze the within-week variations of heart rate, session-rated of perceived exertion (sRPE), total distance, distance in 8.0–11.99 km/h^−1^, recovery distance in 12.0–17.99 km/h^−1^, distance in >18.0 km/h^−1^, maximum speed, number of sprints, heart rate variability, delayed onset muscle soreness (DOMS), and fatigue during training camps of a national futsal team; and (2) to analyze the relationships between load and the well-being. Twenty-eight men from the Chinese Taipei U−20 national futsal team were analyzed. Comparisons of training days revealed that the total distance was significantly smaller on day 1 (d = −1.22) and day 6 (d = −1.95) than on day 3. The sRPE values were significantly lower on day 1 than days 4 (d = −1.53), 5 (d = −2.07), and 6 (d = −2.59). The relationships between training load and recovery parameters revealed moderate correlations between the DOMS and the sRPE recorded one (*r* = −0.321) and two days before training (*r* = −0.289). It is possible conclude that first day imposed a smaller external load and internal load, and that the internal load had a greater dependent relationship with reported DOMS and fatigue during the training camps.

## 1. Introduction

Congested periods of training (e.g., training camps) are often used by national youth teams to employ new dynamics among teammates and also to find or select new players for the final squad [1,2]. Training camps consist of a period (usually one week or more) in which players may be under evaluation by national coaches executing one or more training session per day. Usually, sessions in training camps are field based and mostly tactical/technical related. Small-sided games, positioning games, and regular games are the most common activities in those training camps. Based on the fact that these periods (training camps) are exclusively dedicated to improving the overall team’s dynamics and selecting the players for the final squad, it is expected that a high level of training load will be promoted, mainly considering that in some of the days there may occur two training sessions [3]. This congested calendar may promote changes in the stimulus–recovery dynamics, and, for that reason, it is important to employ a player monitoring cycle that can help coaches and sports scientists properly manage the load and readiness of players [4].

Commonly, players’ monitoring cycles are focused on monitoring external and internal loads, the wellness status of players, and readiness [4]. The external load represents the physical and neuromuscular demands imposed on the players by the tasks and drills, while the internal load represents the biological impact of the external load [5]. Typically, the external load is assessed by global positioning systems (GPSs) or inertial measurement units [6]. The internal load can be assessed both by objective (e.g., heart rate (HR) monitors, blood lactate concentrations) and subjective (e.g., rate of perceived exertion (RPE), effort scales) instruments [7]. However, sports scientists should not only consider the dynamics of load. The possible impact of load on the wellness status of players (and recovery, naturally) should also be considered in a well-implemented player monitoring cycle [8,9].

One of the most common measures used to control the wellness and recovery status in players is HR variability (HRV) [10]. This measure characterizes the parasympathetic and sympathetic influences of the autonomic nervous system on the sinus node by recording the beat-to-beat HR intervals [10]. The HRV can be classified as an objective measure; however, other subjective wellness measures have been proposed and applied in the context of team sports monitoring [11,12]. Among other topics, delayed onset muscle soreness (DOMS), stress, fatigue, sleep quality, and mood are normally assessed in players using questionnaires or scales [13]. Both objective and subjective measures of wellness can provide useful information about the daily variations of players, representing a possible interaction with the stimulus imposed by the training [14].

The research on training load and wellness is progressively growing, mainly in football [15]. Research has also been conducted in futsal [16,17]; however, the evidence is scanty, and some important external load measures have been not reported. In a study that described the perceived training load of futsal players over forty-five weeks, Rabelo et al. [18] showed that the players’ training loads were typically lower than intended by the coach. Also, using perceptive scales of exertion, Clemente et al. [19] compared two types of training weeks (normal versus congested), revealing that perceptive training load, DOMS, and fatigue were significantly greater in normal weeks and that within-week changes occurred in terms of perceptive load.

In an attempt to identify relationships between daily perceived recovery and circumstantial factors related to training load in futsal, sleep quality, or period of the week, Wilke et al. [20] found that neither recovery classification nor prior training load influenced perceived recovery. Even though the aforementioned study focused on the training load and wellness status of futsal players, his approaches require further study. Among potential research topics is how a training camp can influence the training load and wellness of players and what daily changes can occur during these camps. Another is how training load is related to future wellness variations one day and two days after a training session. Finally, it is important to add objective load measures that consider the physical demands of players instead of only the internal load. Such approaches will provide a better understanding of how both dimensions of load (internal and external) can fluctuate throughout a training camp and how external load may concur to influence the wellness fluctuations.

For these reasons, the purpose of this study was twofold: (i) to analyze the daily variations in training load and psychobiological measures during seven-day training camps attended by national futsal teams and (ii) to test the relationships between training load and psychobiological measures during the training camps.

## 2. Materials and Methods

### 2.1. Experimental Approach

This study followed a descriptive research design and a correlational design in which training load measures (both internal and external) and well-being measures were monitored daily in 7 day training camps and tested for its relationships. Players belonging to an under−20 (U−20) national futsal team were monitored daily during six domestic training camps (Table 1). The monitoring process included an analysis of the training load imposed during the sessions (both external and internal) and recovery processes (HRV, DOMS, and fatigue). The weekly protocol consisted in the day 1 of data collection of height, body mass, and skinfolds collected by an expert sports scientist using a calibrated skin folder (Lange Skinfold Caliper, Beta Technology, USA).

Additionally, on each day of the training camp, HRV was measured in the morning, and subjective scores of DOMS and fatigue were collected individually. During the training sessions, players used Polar Team Pro sensors to measure external and internal loads. After each session, the RPE was also collected as an internal load indicator. Comparisons of internal and external loads, HRV, DOMS, and fatigue between training days were carried out. Moreover, correlations between training loads and the HRV, DOMS, and fatigue of the next two days were also tested. All training sessions were conducted in a sports complex at Wufeng University, Taiwan. All players stayed in the campus dormitory during the training camps.

### 2.2. Participants

Twenty-eight male futsal players that were recruited into the Chinese Taipei U−20 national team’s training camps participated in this study voluntarily. Twenty-two field players and six goalkeepers were part of the sample (mean ± standard deviation: age = 18.07 ± 0.73 y; height = 169.57 ± 8.40 cm; body mass = 64.51 ± 12.19 kg). Eleven players were called up from university futsal teams, while seventeen players were called up from senior high school futsal teams. The recruited players undertook regular futsal training sessions (around 6–10 sessions per week) in home teams. The criteria for inclusion were as follows: (i) participants had to take part in all training sessions within the training camp; (ii) participants did not report injuries or illness during the training camp.

The participants were informed about the study design and were familiarized with the protocol. After that, all participants voluntarily signed an informed consent form. The study followed the ethical standards of the Declaration of Helsinki regarding the study of humans, and the study design was approved by the committee of the University of Taipei.

### 2.3. Heart Rate Variability (HRV)

The heart rate variability (HRV) was assessed every morning using an HR monitor (Polar Team Pro©, Polar Electro, Kemple, Finland). The HRV was assessed in a sitting position in the morning, prior to breakfast. Participants sat on chairs in a comfortable position for five minutes which was followed by five minutes of data collection. The data from the HR monitors were exported to the Polar Team Pro web platform, and the raw data was then used for treatment in Kubios HRV analysis software (Premium version 3.2, Kubios, Kuopio, Finland). Medium artefact correction and smoothing priors set at 500 lambda were used for HRV analysis. The natural log of root mean square of successive RR interval differences (LnRMSSD) was calculated in Microsoft Excel 2016 (Microsoft Corporation, Redmond, WA, USA) and was used to evaluate daily cardiac-autonomic functions during the training camps.

### 2.4. Subjective DOMS and FATIGUE

A 5 point Hooper questionnaire [11] was administered every morning (between 08:00 and 08:30). On the 5 point scale, 1 (lowest score) corresponded to the worst state and 5 (highest score) to the best state. The DOMS and fatigue were used for the present data treatment and study. The scores were provided individually, and a visual analogue scale was used to improve the overall accuracy of the answers.

### 2.5. Internal Load

Each player used an HR monitor (Polar Team Pro, Polar Electro, Kemple, Finland) during training sessions to determine the maximal heart rate achieved (HRmax) and the average heart rate (HRav) during the full session. The Borg CR−10 scale was applied as a subjective instrument thirty minutes after the end of the session [21]. The players were asked to answer the question, “How intense was the session?” using a visual analogue scale in which 0 represented “not at all” and 10 represented “extremely intense.” The scores were provided individually and were then registered and multiplied by the duration of the session (in minutes) to calculate the session-RPE (sRPE) which represents the overall load of the session in terms of arbitrary units [22]. Players were previously familiarized with the scale in order to increase the accuracy of the answers.

### 2.6. External Load

The external load during the training sessions was monitored by Polar Team Pro sensors (Polar Team Pro, Polar Electro, Kemple, Finland). The sensor is a microsensor system that encompasses a 3 dimension accelerometer, a gyroscope, and a digital compass with a sample rate of 200 Hz. The maximal distance for recording the signal was 200 meters. The activities profiles included total distance, distance in zone 1 (8.0–11.99 km/h^−1^), recovery distance in zone 2 (12.0–17.99 km/h^−1^), distance in zone 3 (>18.0 km/h^−1^), maximum speed (maxSpeed), and number of sprints (>24.0 km/h^−1^) [3]. The same unit of measurement (GPS) was used by each player to avoid variability.

### 2.7. Statistical Procedures

Data were preliminary tested for normality (*p* > 0.05) and homogeneity (*p* > 0.05) in the SPSS software (version 23.0, IBM, USA). Thereafter, mean and standard deviation were calculated. Training load measures, HRV, and wellness parameters were compared between days of training (D1, D2, D3, D4, D5, D6, D7) using the standardized effect size of Cohen (d) in the form of pairwise comparisons. The magnitudes of the changes were interpreted based on the following thresholds [23]: 0.0–0.2, trivial; 0.2–0.6, small; 0.6–1.2, moderate, 1.2–2.0, large; >2.0, very large. The calculation of standardized effect size was made on Excel spreadsheets properly designed for the process [24]. Correlations between training load measures, HRV, and wellness parameters were tested using the Pearson’s *r* coefficient for a confidence interval of 95%. The magnitudes of the correlations were defined as in Reference [23]: 0.0–0.1, trivial; 0.1–0.3, small; 0.3–0.5, moderate; 0.5–0.7, large; 0.7–0.9, very large; and 0.9–1.0, nearly perfect. The correlations were executed in the SPSS software (version 23.0, IBM, USA).

## 3. Results

Descriptive statistics of training load parameters during the seven days of training camps can be found in Table 2. The greater total distance (10111.0 ± 4878.7 m), distance covered between 12.0 and 17.99 km/h^−1^ (2131.7 ± 1720.3 m), distance covered above 18.0 km/h^−1^ (1056.6 ± 743.8 m), and number of sprints (27.4 ± 22.2 *n*) were covered at day 5 of the training camps. Considering the sRPE, the greatest load occurred at day 2 (1105.7 ± 363.3 A.U.) and the greatest HRmax at day 4 (187.2 ± 13.1 bpm^−1^).

Descriptive statistics of psychobiological parameters related with recovery and wellness can be found in Table 3. The highest InRMSSD (4.2 ± 0.5 log), DOMS (2.9± 0.9 A.U.), and fatigue (3.1± 0.9 A.U.) were found at the first day of the training camps.

The pairwise comparisons of external and internal training load measures can be found in Table 4 and Table 5, respectively. The total distance was largely smaller in the day 1 comparing to day 3 (−35%; d = −1.22) and day 6 (−49.7%; d = −1.95). Distances covered between 8.0 and 11.99 km/h^−1^ were also largely smaller in day 1 comparing to days 3 (−31.9%; d = −1.27), 4 (−35.0%; d = −1.42), 5 (−36.5%; d = −1.5), 6 (−55.8%; d = −2.69), and 7 (−38.2%; d = −1.59). No large differences were found among training days considering the variables of distances covered between 12.0 and 17.99 km/h^−1^ and >18 km/h^−1^, as well as maxSpeed and number of sprints. 

The sRPE was much lower in day 1 comparing to day 4 (−42.3%; d = −1.53), day 5 (−52.4%; d = −2.07), and day 6 (−60.5%; d = −2.59). The sRPE was much greater in day 2 comparing to day 4 (37.0%; d = 1.27) and day 7 (49.5%; d = 1.88). Finally, sRPE was much lower at day 7 comparing to day 3 (−52.4%; d = −1.76). Considering the average HR, it was found that day 1 was much greater than day 2 (11.3%; d = 1.52) and day 3 (11.4%; d = 1.53).

The pairwise comparisons of HRV and wellness parameters can be found in Table 6. No large differences among training days were found in the pairwise comparisons for the InRMMSD, DOMS, and fatigue reported.

**Figure 1:**
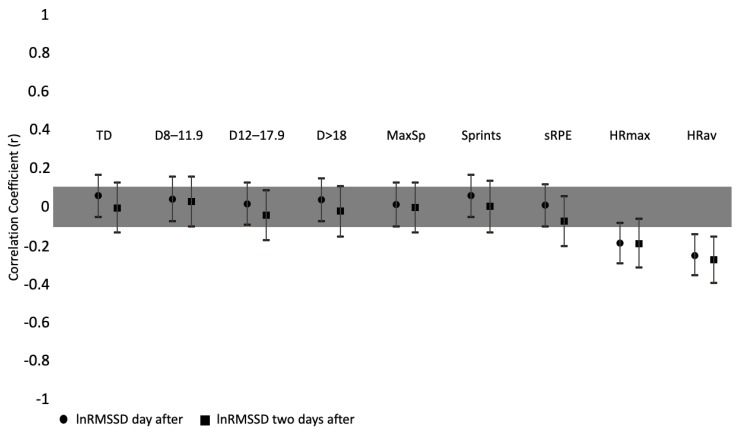
Correlation coefficients between InRMSSD and training load parameters (one day before and two days before). TD: total distance; D: distance at km/h^−1^ intervals; MaxSp: maximum speed; sRPE: session-RPE; HRmax: maximal heart rate; HRav: average heart rate.

Correlations between training load measures (both internal and external) and HRV and wellness parameters were executed. All the valid moments of assessment (sessions per trainin camps) per players were used for the analysis (*n* = 457). The correlations were made between the collected HR and wellness parameters of the day and the training load measures imposed one and two days before. Correlations between InRMSSD and training load can be found in Figure 1. Small but significant correlations were found between the InRMSSD and HRmax of one day before (*r* = −0.186; *p* = 0.001) and two days before (*r* = −0.188; *p* = 0.004) and HRav of one (*r* = −0.250; *p* = 0.000) and two days before (*r* = −0.271; *p* = 0.000).

**Figure 2:**
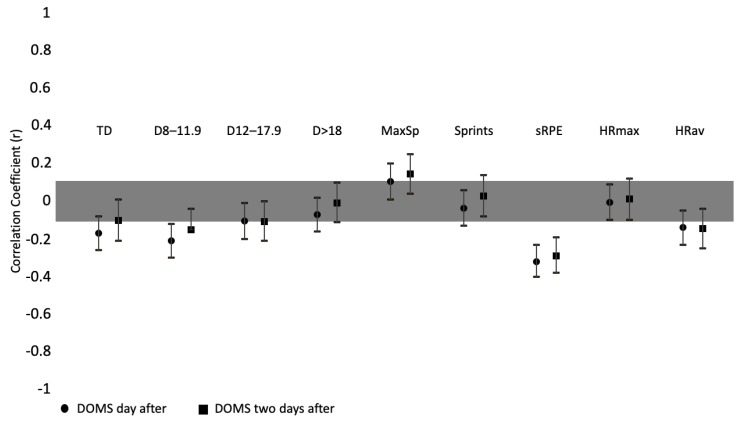
Correlation coefficients between DOMS (delayed onset muscle soreness) and training load parameters (of one day before and two days before). TD: total distance; D: distance at km/h^−1^ intervals; MaxSp: maximum speed; sRPE: session-RPE; HRmax: maximal heart rate; HRav: average heart rate.

Correlations between DOMS and training load can be found in Figure 2. Moderate and significant correlations were found between DOMS and the sRPE of one (*r* = −0.321; *p* < 0.000) and two days before (*r* = −0.289; *p* < 0.000). The DOMS was small but significantly correlated with total distance of one day before (r = −0.170; *p* = 0.000). Significant but small correlations between DOMS and total distance 8.0–11.9 km/h^−1^ of one day (r = −0.210; *p* < 0.000) and two days before the session (*r* = −0.150; *p* = 0.006) were also found. The DOMS was also small but significantly correlated with maximum speed of one (*r* = 0.105; *p* = 0.029) and two days before (*r* = 0.145; *p* = 0.008). Finally, DOMS was small but significantly correlated with average HR of one (*r* = −0.139; *p* = 0.004) and two days before (*r* = −0.145; *p* = 0.008).

**Figure 3:**
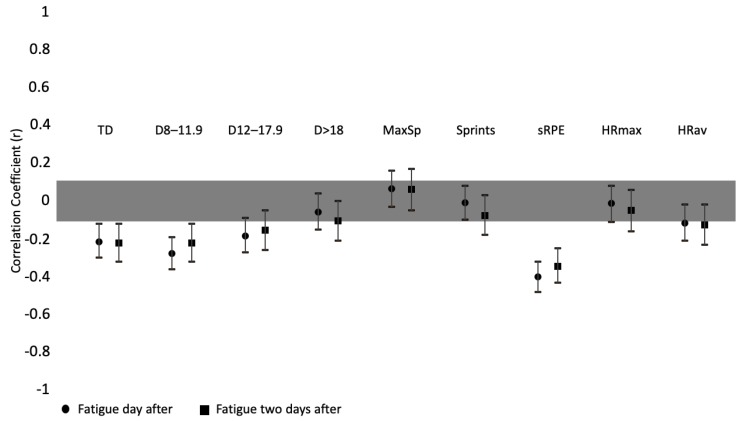
Correlation coefficients between fatigue and training load parameters (of one day before and two days before). TD: total distance; D: distance at km/h^−1^ intervals; MaxSp: maximum speed; sRPE: session-RPE; HRmax: maximal heart rate; HRav: average heart rate.

Correlations between subjective fatigue and training load measures can be found in Figure 3. Moderate correlations were found between fatigue and the sRPE of one (r = −0.401; *p* < 0.000) and two days before (r = −0.344; *p* < 0.000). Fatigue was small but significantly correlated with total distance of one (*r* = −0.214; *p* < 0.000) and two days before (*r* = −0.221; *p* < 0.000).

## 4. Discussion

The present study analyzed the within-week variations of training load measures and wellness parameters and tested possible relationships between the load imposed and the fluctuations in wellness across the week. The main findings revealed that the load on day 1 was significantly smaller than on day 7 in terms of total distance, distances covered between 8.0 and 11.99 km/h^−1^, sRPE, and average HR. On the other hand, the highest HRV and better fatigue perception were found on the first day of the week. Relationships between training load variables and wellness indicators revealed moderate correlations between the load imposed and the DOMS and fatigue reported in the first and second days after the session.

The external load during training sessions has been extensively described in soccer [15]; however, in the context of futsal, such evidence is very limited. Most of the studies that report external load in futsal are centered on match demands [25]. During matches, distances of 100–120 m/min^−1^, total distance in high-intensity running (>15 km/h^−1^) of between 13% and 25%, and total distance in sprinting (>20 km/h^−1^) of between 3% and 9% [26,27] are expected to be observed. Overall, during a futsal match, there can be found values between 319 and 3757 meters covered, and from such distances, 47% is performed at low intensity, 33% at medium intensity, 17% at high intensity, and 4% at maximum intensity [28].

In our study, we monitored the external load during training sessions, and the evidence pointed to distances that varied between 6000 and 10,000 meters, depending on the day. Pairwise comparisons revealed that days 1 and 7 of the training camps contributed to significantly smaller distances compared to the remaining days and that the greatest distances occurred on days 2, 3, 5, and 6 of the seven-day period. Additionally, it was found that distances between 12 and 18 km/h^−1^ varied between 1250 and 2132 meters per session, with the smallest distances occurring on days 1 and 7 and the highest occurring on days 3 and 5. Similarly, distances above 18 km/h^−1^ were the greatest on days 2 (1001 meters) and 5 (1057 meters) and smallest on days 1 (583 meters) and 7 (656 meters). No significant differences among training days were found considering maximal speed. However, the data for the number of sprints per session revealed that the fewest sprints occurred on days 1 and 7 (*n* = 14) and that the greatest number of sprints were observed on days 2 (*n* = 26), 3 (*n* = 25), and 5 (*n* = 27).

Internal load in futsal was researched more than external load. Session-RPE, as an easy-to-use and valid approach to quantifying internal load, is one of the most common measures described in the literature of futsal and training [16,19]. In the study by Moreira et al. [16], values between 6000 and 2000 A.U. per week were found in a four-week analysis of training sessions. In a longitudinal study of a full season, Clemente et al. [19] revealed values of between 338 and 693 A.U. per session. In our study, conducted during training camps, sRPE values of between 609 and 1153 A.U. per day were observed, thus showing the great load imposed during these kinds of periods. The greatest loads occurred on days 2 and 3 of the week (1106 and 1153 A.U., respectively), and the lowest loads occurred on days 1 and 7 (675 and 609 A.U., respectively). This accumulation of load may have some impact on the mechanisms of recovery. We have tested this hypothesis using a correlational approach.

Interestingly, among all the training load measures, sRPE had the strongest correlation with subjective fatigue (reported on the first and second day after the load imposed) and DOMS (also reported on the first and second day after the load imposed). The moderate and significant correlations were inverse, thus suggesting that a greater load was related to a worse perceptive status of fatigue and DOMS. Such evidence is in line with the studies conducted in soccer that revealed moderate correlations between sRPE and fatigue, DOMS, and Hooper’s scores [29]. Similarly, an acute load seems to be moderately to largely correlated with DOMS and Hooper’s score in elite volleyball players [30]. In our study, DOMS was also inversely correlated with total distance, distances covered between 8 and 11.9 km/h^−1^, max speed, and average HR, albeit with a small magnitude. This suggests that sRPE can provide useful information for coaches regarding the relationships among the overall impact of a training session in the perception of recovery. In fact, a meta-analysis dedicated to examining the relationships between internal and external load measures revealed that sRPE has a consistent positive association with external load measures [31].

The present study had some limitations. One of the limitations was that the data came from a single national team, and for that reason, our findings can be used only for descriptive purposes. The training load and the associated mechanism were dependent on this team’s training plans, and for that reason, studies using more than one team are highly recommended. A second limitation was that the values were means of the team; however, variability between subjects should be considered to improve the generalizability of the data. Despite this study’s limitations, this study is one of the few (as far as we know) that describe the external load demands of futsal players during training camps. Moreover, it is also one of the few that analyze the load and recovery mechanisms and attempt to draw connections between them. This provides valuable information to coaches and a new insight on futsal, mainly considering the inverse relationship found between overall load imposed and the perception of DOMS and fatigue of the players.

## 5. Conclusions

Considering the objective of analyzing the daily variations of load and well-being, the main findings of this study revealed that during seven-day training camps, the smallest external and internal loads were imposed on days 1 and 7, while the greatest loads occurred in the middle of the week. In short, it is possible to conclude that the first and last days of training camps imposed a significantly smaller external load on players (in different intensities) and that the middle of the week was more dedicated to increasing the overall volume of physical demands. No within-week changes were found in terms of HRV, DOMS, or fatigue. Considering the second objective of the study (analyze the relationships between load and well-being measures, the main findings revealed that the DOMS and fatigue were moderately and inversely correlated with sRPE as reported one and two days before the camp.

## Figures and Tables

**Table 1:** Description of the number of sessions and time of sessions per day during the included training camps.

	Period	*n* of Players	D1		D2		D3		D4		D5		D6		D7	
			Min	Ns	Min	Ns	Min	Ns	Min	Ns	Min	Ns	Min	Ns	Min	Ns
TC_1_	July 9th to 15th 2018	21	116	1	116	1	115	1	121	1	225	2	131	1	113	1
TC_2_	September 1st to 7th 2018	23	-	1	238	2	260	2	131	1	250	2	277	2	140	1
TC_3_	September 21st to 27th 2018	17	-	1	243	2	270	2	131	1	254	2	217	2	98	1
TC_4_	October 5th to 11th 2018	19	72	1	167	2	199	2	217	2	258	3	110	1	123	1
TC_5_	October 18th to 24th 2018	16	110	1	213	2	197	2	253	2	109	1	228	2	83	1
TC_6_	November 5th to 11th 2018	17	136	1	218	2	242	2	98	1	239	2	216	2	121	1

Min: minutes of training in that day; Ns: number of sessions in that day; TC1: first training camp; TC2: second training camp; C3: third training camp; TC4: fourth training camp; TC5: fifth training camp; TC6: sixth training camp.

**Table 2:** Descriptive statistics of training load parameters during the different days of training camps.

	Day 1	Day 2	Day 3	Day 4	Day 5	Day 6	Day 7
Total distance (m)	6830.4 ± 2142.7	9257.7 ± 4075	9400.8 ± 3980.1	7964.4 ± 3366.6	10111.0 ± 4878.7	9170.7 ± 4585.3	6191.8 ± 1715.0
D8.0–11.99 km/h^−1^ (m)	1304.6 ± 335.7	1617.4 ± 819.0	1840.0 ± 1197.4	1325.2 ± 619.4	1742.3 ± 1263.4	1566.3 ± 882.7	1015.6 ± 330.1
D12.0–17.99 km/h^−1^ (m)	1250.1 ± 405.8	1851.5 ± 967.5	2080.3 ± 1336.3	1623.0 ± 848.9	2131.7 ± 1720.3	1846.2 ± 1020.6	1351.08 ± 499.3
D > 18.0 km/h^−1^ (m)	582.9 ± 304.2	1000.5 ± 690.3	999.0 ± 709.4	829.7 ± 665.3	1056.6 ± 743.8	899.9 ± 574.1	655.5 ± 394.3
MaxSpeed (km/h^−1^)	28.0 ± 3.3	27.2 ± 2.5	27.2 ± 4.7	27.6 ± 3.3	27.8 ± 3.2	28.1 ± 2.6	28.1 ± 3.3
Sprints (*n*)	14.3 ± 12.2	25.7 ± 21.8	25.1 ± 21.7	21.2 ± 21.9	27.4 ± 22.2	22.8 ± 18.7	14.4 ± 12.4
sRPE (A.U.)	674.5 ± 251.0	1105.7 ± 363.3	1153.3 ± 433.3	772.5 ± 441.4	998.6 ± 491.6	838.4 ± 393.5	609.0 ± 187.8
HRmax (bpm^−1^)	187.3 ± 10.8	185.0 ± 12.2	185.0 ± 14.5	187.2 ± 13.1	183.1 ± 10.6	184.9 ± 11.9	184.9 ± 12.2
HRav (bpm^−1^)	139.8 ± 10.5	134.2 12.9	130.7 ± 13.8	132.5 13.3	127.9 12.4	131.0 11.0	130.0 ± 11.8

D: distance; A.U.: arbitrary units; bpm^−1^: beats per minute; *n*: number; m: meters; km/h^−1^: kilometers per hour; HRmax: maximal heart rate; HRav: average heart rate; MaxSpeed: maximal speed; *n*: number; sprints: >24.0 km/h^−1.^

**Table 3:** Descriptive statistics of psychobiological parameters during the different days of training camps.

	Day 1	Day 2	Day 3	Day 4	Day 5	Day 6	Day 7
InRMSSD (log)	4.2 ± 0.5	3.9 ± 0.5	4.0 ± 0.5	3.8 ± 0.6	3.9 ± 0.6	3.9 ± 0.5	3.9 ± 0.5
DOMS (A.U.)	2.9 ± 0.9	2.8 ± 0.7	2.6 ± 0.8	2.5 ± 0.8	2.6 ± 0.8	2.6 ± 0.7	2.6 ± 0.7
Fatigue (A.U.)	3.1 ± 0.9	2.6 ± 0.6	2.4 ± 0.7	2.3 ± 0.6	2.4 ± 0.6	2.4 ± 0.6	2.5 ± 0.7

InRMSSD: natural log of root mean square of successive RR interval differences; A.U.: arbitrary units; DOMS: delayed onset muscle soreness.

**Table 4:** Pairwise comparisons (standardized differences of Cohen (95% confident interval)) of external training load measures between training days (D).

**Total Distance**	**D2**	**D3**	**D4**	**D5**	**D6**	**D7**
D1	−0.48 [−1.0; 0.0]	−1.22 [−0.2; −0.6]	−0.97 [−1.8; −0.2]	−1.1 [−2.1; −0.1]	−1.95 [−2.6; −1.3]	−0.34 [−0.7; −0.0]
D2		−0.03 [−0.2; 0.2]	−0.45 [−0.6; −0.3]	−0.09 [−0.4; 0.2]	−0.21 [−0.5; 0.0]	−0.72 [−1.0; −0.5]
D3			−0.40 [−0.6; −0.2]	0.03 [−0.2; 0.3]	−0.10 [−0.3; 0.1]	−0.8 [−1.0; −0.6]
D4				0.37 [0.1; 0.6]	0.12 [−0.2; 0.4]	−0.54 [−0.8; −0.3]
D5					−0.26 [−0.5; −0.0]	−1.02 [−1.2; −0.9]
D6						−0.7 [−0.9; −0.6]
**Distances 8.0−11.99 km.h^−1^ (m)**	**D2**	**D3**	**D4**	**D5**	**D6**	**D7**
D1	−0.70 [−1.3; −0.1]	−1.27 [−1.9; −0.6]	−1.42 [−2.3; −0.5]	−1.5 [−2.5; −0.5]	−2.69 [−3.2; −2.2]	−1.59 [−2.0; −1.2]
D2		0.13 [−0.1; 0.3]	−0.53 [−0.8; −0.3]	−0.27 [−0.6; 0.0]	−0.29 [−0.6; −0.0]	−0.85 [−1.1; −0.6]
D3			−0.45 [−0.6; −0.3]	−0.15 [−0.4; 0.1]	−0.18 [−0.4; 0.1]	−0.69 [−0.9; −0.5]
D4				0.26 [0.0; 0.5]	0.12 [−0.2; 0.4]	−0.48 [−0.8; −0.2]
D5					−0.17 [−0.4; 0.1]	−0.81 [−1.0; −0.6]
D6						−0.63 [−0.8; −0.5]
**Distances 12.0−17.99 km.h^−1^ (m)**	**D2**	**D3**	**D4**	**D5**	**D6**	**D7**
D1	−0.43 [−1.0; 0.2]	−0.87 [−1.6; −0.1]	−0.90 [−1.7; −0.1]	−1.05 [−2.0; −0.1]	−1.75 [−2.3; −1.2]	−1.09 [−1.7; −0.5]
D2		0.03 [−0.2; 0.2]	−0.52 [−0.7; −0.3]	−0.24 [−0.5; 0.0]	−0.25 [−0.5; 0.0]	−0.61 [−0.8; −0.4]
D3			−0.33 [−0.5; −0.2]	−0.06 [−0.3; 0.2]	−0.07 [−0.3; 0.1]	−0.4 [−0.6; −0.2]
D4				0.21 [−0.02; 0.4]	0.11 [−0.1; 0.3]	−0.21 [−0.4; 0.0]
D5					−0.14 [−0.4; 0.1]	−0.62 [−0.8; −0.5]
D6						−0.51 [−0.7; −0.3]
**Distances >18.0 km.h^−1^ (m)**	**D2**	**D3**	**D4**	**D5**	**D6**	**D7**
D1	−0.60 [−1.4; 0.1]	−0.60 [−1.2; −0.0]	−0.48 [−1.1; 0.1]	−1.10 [−1.8; −0.4]	−0.21 [−0.7; 0.3]	−0.48 [−1.0; 0.0]
D2		−0.11 [−0.2; 0.01]	−0.46 [−0.6; −0.3]	−0.18 [−0.4; −0.0]	−0.14 [−0.3; 0.0]	−0.41 [−0.6; −0.3]
D3			−0.37 [−0.6; −0.2]	−0.09 [−0.3; 0.1]	−0.09 [−0.2; 0.1]	−0.49 [−0.6; −0.4]
D4				0.21 [−0.0; 0.4]	0.16 [−0.0; 0.4]	−0.08 [−0.3; 0.1]
D5					−0.04 [−0.2; 0.1]	−0.52 [−0.7; −0.4]
D6						−0.60 [−0.8; −0.5]
**Maximum speed (km/h^−1^)**	**D2**	**D3**	**D4**	**D5**	**D6**	**D7**
D1	−0.44 [−0.8; −0.1]	0.13 [−0.3; 0.6]	0.07 [−0.1; 0.2]	−0.22 [−0.6; 0.1]	0.57 [0.1; 1.1]	−0.81 [−1.7; 0.1]
D2		−0.14 [−0.4; 0.1]	0.03 [−0.2; 0.2]	0.15 [−0.1; 0.4]	0.26 [0.1; 0.4]	0.24 [−0.0; 0.5]
D3			0.10 [−0.0; 0.2]	0.20 [0.0; 0.4]	0.23 [0.1; 0.4]	0.32 [0.1; 0.5]
D4				0.09 [−0.1; 0.3]	0.17 [−0.0; 0.4]	0.17 [−0.1; 0.4]
D5					0.16 [0.0; 0.3]	0.07 [−0.1; 0.3]
D6						0.12 [−0.1; 0.4]
**Sprints (n)**	**D2**	**D3**	**D4**	**D5**	**D6**	**D7**
D1	−0.04 [−0.4; 0.3]	−0.08 [−0.4; 0.2]	−0.18 [−0.6; 0.2]	−0.44 [−1.0; 0.1]	0.27 [−0.2; 0.7]	0.11 [−0.4; 0.6]
D2		−0.05 [−0.2; 0.1]	−0.47 [−0.7; −0.3]	−0.04 [−0.2; 0.2]	−0.14 [−0.3; 0.0]	−0.54 [−0.7; −0.4]
D3			−0.36 [−0.6; −0.2]	0.04 [−0.1; 0.2]	−0.08 [−0.3; 0.1]	−0.62 [−0.8; −0.5]
D4				0.29 [0.1; 0.5]	0.12 [−0.1; 0.3]	−0.26 [−0.5; 0.0]
D5					−0.13 [−0.3; 0.0]	−0.78 [−1.0; −0.6]
D6						−0.65 [−0.8; −0.5]

Legend: D1: day 1; D2: day 2; D3: day 3; D4: day 4; D5: day 5; D6; day 6; D7: day 7; A.U.: arbitrary units; *n*: number; m: meters; km/h^−1^: kilometers per hour; MaxSpeed: maximal speed; *n*: number; sprints: >24.0 km/h^−1^.

**Table 5:** Pairwise comparisons (standardized differences of Cohen (95% confident interval)) of internal training load measures between training days (D).

**sRPE (A.U.)**	**D2**	**D3**	**D4**	**D5**	**D6**	**D7**
D1	0.45 [0.1; 0.8]	−0.37 [−0.7; 0.0]	−1.53 [−2.0; −1.1]	−2.07 [−3.0; −1.1]	−2.59 [−3.3; −1.9]	0.29 [0.2; 0.4]
D2		−0.02 [−0.2; 0.1]	−1.27 [−1.5; −1.01]	−0.78 [−1.1; −0.5]	−1.08 [−1.4; −0.8]	−1.88 [−2.0; −1.7]
D3			−1.07 [−1.3; −0.9]	−0.68 [−0.9; −0.5]	−0.90 [−1.2; −0.7]	−1.76 [−1.9; −1.6]
D4				0.33 [0.1; 0.6]	0.08 [−0.2; 0.3]	−0.56 [−0.8; −0.3]
D5					−0.16 [−0.3; −0.0]	−0.78 [−0.9; −0.7]
D6						−0.76 [−0.9; −0.6]
**HRmax (bpm^−1^)**	**D2**	**D3**	**D4**	**D5**	**D6**	**D7**
D1	−0.91 [−1.5; −0.3]	−0.22 [−1.3; 0.8]	0.03 [−0.3; 0.4]	−0.38 [−0.9; 0.1]	−0.23 [−0.6; 0.2]	−0.69 [−2.2; 0.8]
D2		−0.15 [−0.4; 0.1]	−0.05 [−0.2; 0.1]	−0.32 [−0.5; −0.1]	−0.09 [−0.3; 0.1]	0.01 [−0.2; 0.3]
D3			0.03 [−0.2; 0.2]	−0.17 [−0.4; 0.0]	−0.01 [−0.3; 0.2]	0.11 [−0.2; 0.4]
D4				−0.35 [−0.6; −0.2]	−0.17 [−0.4; 0.0]	−0.05 [−0.3; 0.2]
D5					0.09 [−0.1; 0.3]	0.02 [−0.2; 0.2]
D6						0.07 [−0.1; 0.3]
**HRav (bpm^−1^)**	**D2**	**D3**	**D4**	**D5**	**D6**	**D7**
D1	−1.52 [−1.9; −1.1]	−1.53 [−2.4; −0.7]	−0.93 [−1.8; −0.1]	−0.60 [−0.9; −0.3]	−0.45 [−0.7; −0.2]	−0.97 [−1.7; −0.2]
D2		−0.47 [−0.6; −0.3]	−0.34 [−0.5; −0.2]	−0.71 [−0.9; −0.5]	−0.29 [−0.5; −0.1]	−0.36 [−0.6; −0.1]
D3			0.02 [−0.1; 0.2]	−0.32 [−0.5; −0.1]	0.01 [−0.2; 0.2]	0.02 [−0.2; 0.3]
D4				−0.45 [−0.6; −0.3]	−0.10 [−0.3; 0.1]	−0.60 [−3.3; 2.3]
D5					0.19 [0.0; 0.4]	0.16 [−0.1; 0.4]
D6						−0.70 [−2.8; 1.3]

Legend: D1: day 1; D2: day 2; D3: day 3; D4: day 4; D5: day 5; D6; day 6; D7: day 7; sRPE: session-RPE; HRmax: maximal heart rate; HRav: average heart rate; A.U.: arbitrary units; bpm^−1^: beats per minute.

**Table 6:** Pairwise comparisons (standardized differences of Cohen (95% confident interval)) of HR variability and wellness parameters between training days (D).

**InRMMSD**	**D2**	**D3**	**D4**	**D5**	**D6**	**D7**
D1	−0.21 [−0.5; 0.0]	−0.03 [−0.2; 0.1]	0.80 [0.5; 1.1]	0.02 [−0.1; 0.1]	−0.31 [−0.8; 0.2]	−0.12 [−0.5; 0.2]
D2		0.09 [−0.1; 0.2]	−0.12 [−0.4; 0.1]	0.08 [−0.1; −0.2]	−0.04 [−0.3; 0.2]	−0.01 [−0.3; 0.2]
D3			−0.23 [−0.5; 0.0]	−0.10 [−0.3; 0.1]	−0.13 [−0.3; 0.1]	−0.05 [−0.3; 0.1]
D4				0.08 [−0.1; 0.3]	0.10 [−0.1; 0.3]	0.18 [−0.1; 0.5]
D5					0.07 [−0.1; 0.2]	0.09 [−0.1; 0.3]
D6						0.09 [−0.1; 0.3]
**DOMS**	**D2**	**D3**	**D4**	**D5**	**D6**	**D7**
D1	−0.07 [−0.5; 0.4]	−0.30 [−0.9; 0.3]	0.50 [0.3; 0.7]	−0.09 [−0.8; 0.6]	0.08 [−0.4; 0.6]	−0.14 [−0.6; 0.3]
D2		−0.28 [−0.5; −0.1]	−0.38 [−0.6; −0.2]	−0.37 [−0.6; −0.2]	−0.32 [−0.5; −0.1]	−0.28 [−0.5; −0.1]
D3			−0.14 [−0.3; 0.0]	−0.12 [−0.3; 0.1]	−0.06 [−0.2; 0.1]	−0.01 [−0.2; 0.2]
D4				0.08 [−0.1; 0.3]	0.10 [−0.1; 0.3]	0.18 [−0.1; 0.5]
D5					0.07 [−0.1; 0.2]	0.09 [−0.1; 0.3]
D6						0.09 [−0.1; 0.3]
**Fatigue**	**D2**	**D3**	**D4**	**D5**	**D6**	**D7**
D1	−0.27 [−0.8; 0.2]	−0.52 [−1.1; 0.0]	0.99 [0.3; 1.7]	−0.38 [−0.9; 0.2]	−0.12 [−0.6; 0.4]	0.07 [−0.4; 0.5]
D2		−0.41 [−0.6; −0.2]	−0.60 [−0.8; −0.4]	−0.41 [−0.6; −0.2]	−0.26 [−0.5; −0.1]	−0.16 [−0.4; 0.1]
D3			−0.20 [−0.4; −0.0]	−0.09 [−0.3; 0.1]	0.05 [−0.1; 0.2]	0.15 [−0.0; 0.3]
D4				0.13 [−0.0; 0.3]	0.18 [−0.0; 0.4]	0.27 [0.1; 0.5]
D5					0.11 [−0.0; 0.3]	0.21 [0.1; 0.4]
D6						0.09 [−0.1; 0.2]

Legend: D1: day 1; D2: day 2; D3: day 3; D4: day 4; D5: day 5; D6; day 6; D7: day 7; InRMSSD: natural log of root mean square of successive RR interval differences; A.U.: arbitrary units; DOMS: delayed onset muscle soreness.
